# Transactions Medical Association of Missouri

**Published:** 1852-10

**Authors:** 


***Transactions of the Medical Association of the State of Missouri,*** vol. 2.

THE multiplication of these transactions of state societies show the extending organization of the Medical Profession and excite some hope of permanent good. Is it not time for the state of Maryland to bring forward her medical society? The medical and chirurgical faculty is an old fossil; it has no more vitality than the skin of one of the last seventeen year locusts.

We notice in these transactions a good essay on medical education by Dr. Hammer. The views of the writer are sensible views, and one day will be practicable. In the present, the money changers have possession of the temple.

